# Exploring the Value of Continuous Plantar Temperature Monitoring for Diabetic Foot Health Management: Observational, Prospective Cohort Study

**DOI:** 10.2196/73187

**Published:** 2025-09-12

**Authors:** Maryam Hajizadeh, Emily Matijevich, Emily Bray, Evan Minty, Brock Liden

**Affiliations:** 1 Orpyx Medical Technologies, 1240 20 Ave SE Suite 205, Calgary, AB, T2G 1M8, Canada, 1 5146638551; 2 Department of Medicine, University of Calgary, Calgary, AB, Canada; 3 Cutting Edge Research LLC, Circleville, OH, United States

**Keywords:** continuous temperature monitoring, wearables, sensor-based monitoring, diabetic foot ulcer, temperature, remote patient monitoring

## Abstract

**Background:**

Diabetic foot ulcers (DFUs) are a life-changing complication of diabetes. There is increasing evidence that remote plantar temperature monitoring can reduce the recurrence of DFUs. Monitoring of foot temperature once a day is the current guideline for identifying early signs of foot inflammation. However, single readings of physiological signals can increase the risk of misdiagnosis when the signals fluctuate throughout the day.

**Objective:**

The aim of this study was to evaluate whether intraday temperature asymmetry signals were stable or varied as a function of time in individuals at risk of DFUs.

**Methods:**

In total, 64 participants with diabetes (mean age 68, SD 13.8 y) were provided with multimodal sensory insoles (Orpyx Sensory Insoles) to monitor continuous temperature data at a frequency of once per minute at 5 plantar locations in a 90-day study window. The augmented Dickey-Fuller test was used to determine whether the temperature asymmetry signals were stationary (ie, indicating constant mean and variance over time) or nonstationary (ie, indicating time-varying behaviors or trends in the signal).

**Results:**

The study included 43 participants, 1080 data days, and 5400 contralateral temperature asymmetry signals. Most (4428/5400, 82%) of the temperature asymmetry signals were nonstationary, with intraday fluctuations likely influenced by physiological and environmental factors. Of the nonstationary signals, nearly half (1948/4428, 44%) fluctuated above and below the concerning asymmetry threshold of 2.2 °C. The intraday variability underscores the potential for false-positive and false-negative hot spot detection with once-daily measurements. Substantial variability was observed in stationarity patterns both within and across participants. Notably, concerning asymmetries in nonstationary signals occurred at different time points across participants, measurement windows, and days.

**Conclusions:**

Our findings highlight the value of continuous plantar temperature monitoring for diabetic foot health management, relative to once-daily measurements. Several repeated measurements throughout the day increase confidence with regard to the accuracy of observed plantar physiology trends. Continuous monitoring may improve the accuracy of plantar temperature measurement, unlock new diagnostic capabilities, and support personalized care.

## Introduction

### Background

Diabetic foot ulcers (DFUs) are open wounds on the foot caused by neuropathy, poor circulation, or repetitive stress to the foot tissue [1]. DFUs are a devastating foot complication in individuals with diabetes, with 1 in 5 ulcers resulting in severe infection and amputation [2]. Fortunately, 75% of DFUs are preventable when the risk factors are detected and treated early [3]. Remote plantar temperature monitoring has been proposed as a preferred method for DFU prevention, as tissue inflammation (indicated by warmth) can precede wound formation [2].

Current international guidelines suggest once-daily foot temperature monitoring to identify early signs of foot inflammation [3,4]. Once-daily plantar temperature measurements can be captured with handheld thermometry [5] or with sensor-based digital health technologies (eg, sensory insoles [6], smart mats [7], or sensor-embedded socks [8]). Daily temperature asymmetry measures (difference between similar regions on 2 feet) are typically compared to an established asymmetry threshold of 2.2 °C to identify a trend of concern (ie, a temperature hot spot) [7-10]. While studies informing once-daily plantar temperature measurements have shown most success in DFU risk reduction when managed with marked reductions in activity [5], there are concerns that once-daily plantar temperature measurements may not provide a comprehensive view of foot temperature dynamics. Once-daily measurements may also lack the accuracy needed to limit false negatives (which could result in missed, concerning trends) or false positives (which could unnecessarily recommend sedentary behavior in a diabetic population). Key fluctuations in temperature asymmetry that indicate early-stage complications may occur outside the once-daily measurement [11,12].

Single readings of health measurements increase the risk of misdiagnoses due to false-positive and false-negative test results. Many physiological signals fluctuate throughout the day as a function of behavior, activity, environment, circadian rhythms, and other factors [13-15]. While single health measurements remain valuable in some contexts, repeated or continuous measurements are now being taken to improve accuracy. For example, rather than a single blood pressure measurement, at least 4 daily home blood pressure measurements over 2 days are recommended to accurately diagnose out-of-clinic hypertension [14]. For glycemic management, continuous glucose monitoring has become a widely adopted supplement to intermittent capillary blood glucose testing as it more accurately reflects how lifestyle factors impact glucose fluctuations, reducing the risk of hypo- and hyperglycemia events [16,17]. For outpatient fever monitoring in high-risk patients, continuous temperature monitoring, rather than daily oral temperature assessment, has been shown to detect fever onset more accurately and with greater lead time [18].

Wearable devices that facilitate continuous plantar temperature monitoring have shown that absolute plantar temperatures can vary as a function of foot and body posture, limb perfusion, activity levels, disease status, and other factors [12,19]. For example, Beach et al [12] used a wearable device for continuous plantar foot temperature monitoring to calculate dynamic temperature parameters (eg, rise time) in the sitting and standing positions in participants with diabetes and healthy controls. Foot temperature rise times were significantly higher in participants with diabetes, potentially due to differences in soft tissue biomechanics and vascularization. The authors concluded, “temperature monitoring for DFUs focuses on a single snapshot of temperature differences, which may not represent the more complex dynamics of temperature change present in those with diabetes” [12].

### Objective

It remains unknown if plantar temperature *asymmetries*, the key biomarker in remote temperature monitoring for DFU management protocols, vary throughout the day. The aim of this analysis was to evaluate whether intraday plantar temperature asymmetries vary as a function of time in individuals with diabetes and a history of DFUs.

## Methods

### Study Design

The following sections describe a secondary analysis of a broader clinical study evaluating the use of the Orpyx Sensory Insole (Orpyx Medical Technologies Inc.) system as an adjunct to diabetic peripheral neuropathy standard of care. The primary outcome of the broader clinical study was to observe the clinical advantages of the sensory insoles alongside the standard of care. This study was reported in accordance with the STROBE (Strengthening the Reporting of Observational Studies in Epidemiology) guidelines (Checklist 1).

### Participants

In total, 64 participants with type 1 or 2 diabetes, peripheral neuropathy, and a history of a previous plantar foot ulcer (mean age 68, SD 13.8 y) were enrolled through a prospective, observational cohort study. Participants were recruited from a single podiatry clinic in Ohio, United States, from April 2022 to January 2023. The exclusion criteria included the presence of active ulcers or other open chronic wounds, severe vascular disease (defined as an ankle-brachial index [ABI] of ≤0.6 or an ABI of ≥1.2, or adequate lower extremity perfusion per physician discretion), a history of nonneuropathic foot ulcers (ie, arterial or venous insufficiency ulcers), or significant balance disorders.

### Technical Implementation

Custom sensory insoles (Orpyx Sensory Insole) were used for data collection. The Orpyx Sensory Insole is a standard-of-care, custom orthotic combined with a technology layer that enables patient-facing biofeedback and remote patient monitoring services.

The custom insoles comprised a custom contoured layer manufactured using a direct milling CAD/CAM (computer-aided design/computer-aided manufacturing) process. When requested by the provider, the insoles were then further modified to accommodate any foot abnormalities. The insole materials and fabrication process meet HCPCS A5514 requirements.

**Figure 1.**
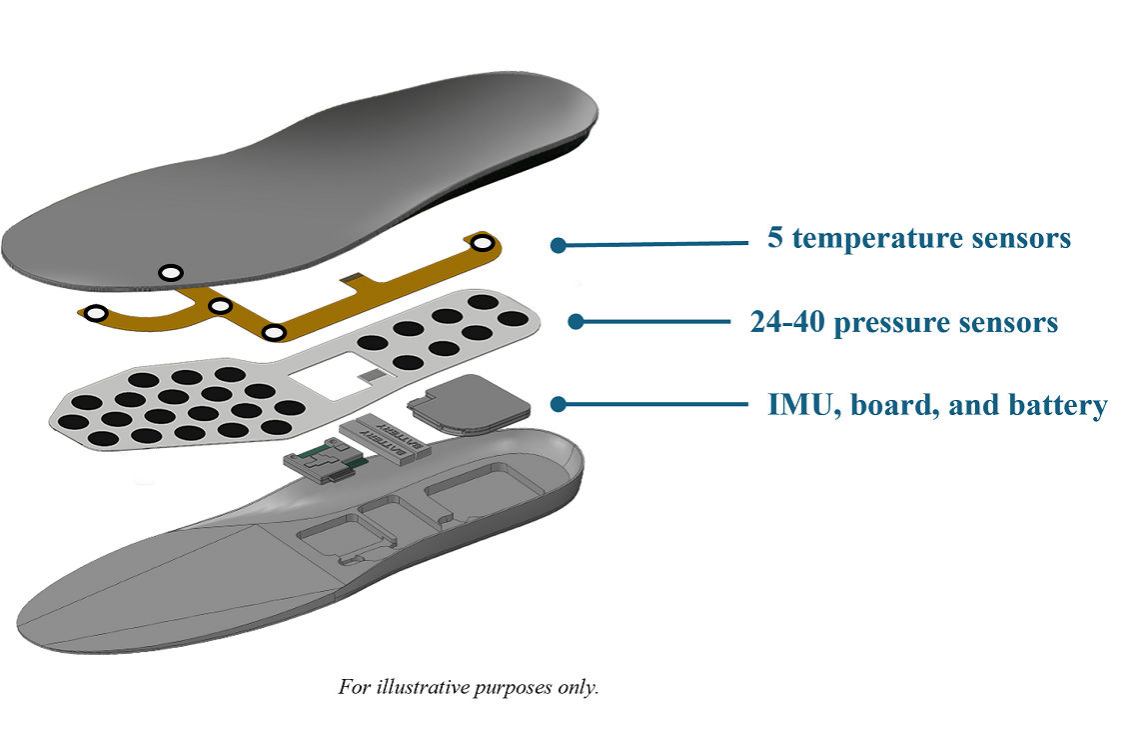
The multimodal Orpyx Sensory Insole system includes custom-milled insoles that are placed into the participants’ shoes. The insoles include a technology layer to enable multimodal monitoring and biofeedback to participants and providers. The technology layer includes temperature sensors, pressure sensors, and an inertial measurement unit (IMU) sensor. For this analysis, only temperature data measured by the 5 temperature sensors were used.

The technology layer included multiple sensors and electronics that enable multimodal, continuous, underfoot data collection (Figure 1). Depending on the size of the insole, the technology layer contains between 24 and 40 pressure sensors. In addition, 5 temperature sensors are embedded and positioned at the big toe; metatarsal heads 1 (Meta 1), 3 (Meta 3), and 5 (Meta 5); and the heel (the areas of bony prominence) that are the most frequent locations for DFU formation. Finally, the electronics assembly includes an inertial measurement unit (IMU) that enables the ability to track steps and wear time.

### Data Collection and Analysis

Participants wore the sensory insoles for up to 90 days in standardized diabetic footwear, with a recommended daily adherence of 4.5 hours per day [20]. Participation in the study concluded at the end of the 90-day period, upon the development of a plantar foot ulcer within the monitoring window or if the participant withdrew for another reason. Participants were also provided remote patient monitoring services through the in-house remote patient monitoring service at Orpyx Medical Technologies. A case series, focused on the value of multimodal sensing across multiple physiological domains for managing diabetic foot health, was previously published for 3 participants from this study cohort [21].

Plantar temperature data, logged at a frequency of once per minute, were the only parameter used for this analysis. The daily contralateral temperature asymmetry time-series signal was calculated as the difference between continuous temperatures at the corresponding left and right foot regions. Therefore, each sensory insole pair provided 5 temperature asymmetry signals. A series of data-cleaning steps corrected the underlying signal bias and masked portions of the time-series signals that were unreliable measures of foot skin temperature (eg, periods surrounding insole donning and doffing). The remaining signals were smoothed using a rolling window mean (window size=10 minutes).

For this analysis, participants were excluded if they withdrew from the study before insole dispensing, withdrew after minimal device use (defined as no data day with a temperature asymmetry measurement >2 h), or were enrolled as unilateral users, as these participants did not generate plantar temperature asymmetry data. Participants with previous minor lower-limb amputations (eg, removal of the first digit) were also excluded from this specific analysis to control for the potential impact of missing foot anatomy on temperature asymmetry signals. Furthermore, short-duration temperature asymmetry measurements (<2 h) were excluded from this analysis. The short-duration measurement exclusion criteria were specifically to accommodate this exploratory statistical analysis and were more rigorous than what were used in our remote temperature monitoring protocol for DFU management.

### Statistical Analysis and Outcomes

A stationarity test was conducted to assess whether the time-series temperature asymmetry signals remained constant over time. Specifically, we used the augmented Dickey-Fuller (ADF) test, performed in Python (version 3.10) with the adfuller function from the statsmodels Python package (version 0.13.2), to evaluate the stationarity of each daily temperature asymmetry time series [22,23]. The ADF test evaluates the null hypothesis that a unit root is present in the time series, indicating nonstationarity due to a stochastic trend [23,24]. In our analysis, we specified regression=“c” to assume the data may have a nonzero mean but no trend term. Maximum lag was not manually defined (maxlag=None), while the test automatically selected the optimal number of lags based on the Akaike information criterion (AIC) by setting autolag=“AIC,” which balances model fit and complexity. The test yields a test statistic, which is compared against critical values from simulated distributions to assess statistical significance. A *P* value ≤.05 was considered evidence against the null hypothesis, indicating that the time series is “weakly stationary.” This infers that certain statistical properties, such as mean, variance, and autocovariance remain constant over that wear period. For this paper, we refer to contralateral temperature time series from the data days in which the ADF test rejects the null hypothesis at a *P* value threshold ≤.05 as “stationary” and in which the ADF test fails to reject the null hypothesis at a *P* value threshold >.05 as “nonstationary.”

The stationarity result of all daily signals was aggregated for each participant to calculate the percentage of stationary versus nonstationary signals over their adherence days in the 90-day study period. Individual data days were reviewed to determine if the signals at all 5 plantar locations were consistently stationary, consistently nonstationary, or a mixed result (some stationary and others nonstationary).

Given that nonstationary temperature asymmetry signals will exhibit trend and variability, further analysis was conducted to determine the proportion of measurements within a day that were symmetric versus asymmetric. As no established threshold currently exists for defining concerning asymmetry in *continuous* temperature monitoring, the established threshold for once-daily temperature monitoring (2.2 °C) was used [7-10]. For nonstationary signals with one or more temperature asymmetry measurements exceeding this threshold, the percentage of intraday measurements with “concerning asymmetry” (defined as asymmetry >2.2 °C) was calculated, similar to how continuous glucose measurements are classified as “time in range” and “time above or below range” [17].

Finally, nonstationary signals with at least one concerning asymmetry measurement were qualitatively examined to determine the time of day at which these concerning asymmetrical measurements occurred. This analysis provided insights into whether periods of concerning asymmetry were consistent and repeatable or temporally dispersed between days and across participants.

### Ethical Considerations

This study was approved by the WCG Institutional Review Board (20220828) on October 3, 2022. Informed consent was obtained from all participants. The original informed consent permitted secondary analyses without requiring additional consent from participants. Participant data were stored on secure, access-controlled servers. Data were deidentified for this analysis and only accessible by authorized research personnel. Participants did not receive any financial compensation for participation in this study.

## Results

### Participant Characteristics

**Figure 2.**
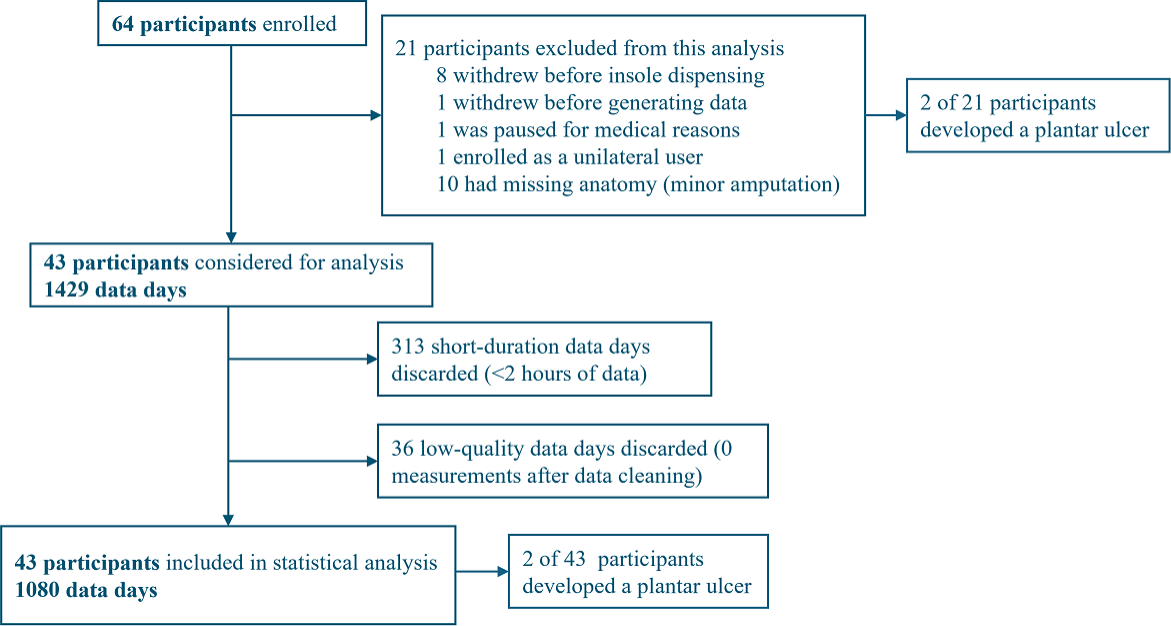
Flowchart of the process used in selecting participants and data days for inclusion in the study analysis.

A total of 43 participants were included in this analysis (Table 1; Figure 2), of which 21 participants were excluded: 8 participants withdrew from the study before insole dispensing, 2 participants withdrew after minimal device use, 1 participant enrolled as a unilateral user, and 10 participants had previous minor lower-limb amputations.

Across the 43 participants, 1429 data days with temperature asymmetry measurements were considered for further data cleaning and analysis (Figure 2); 313 short-duration data days (<2 h) were discarded, in addition to 36 low-quality data days that had no remaining measurements after data cleaning (Figure 2). Ultimately, 43 participants and 1080 data days provided 5400 temperature asymmetry signals for statistical analysis (Figure 2).

Across the 64 participants enrolled in this study, 4 participants developed a plantar ulcer within the 90-day study period. Of the 4 participants who ulcerated, two were in a cohort excluded from this analysis and two were in the included cohort.

**Table 1.** Participant characteristics.

Characteristics	Participants (n=43)
Sex, n (%)	
Male	25 (58)
Female	18 (41)
Age (years)	
Values, mean (SD)	68 (13.8)
Values, median (range; IQR)	70 (39-92; 56-77)
Race, n (%)	
Black or African American	1 (13)
White	42 (97)
Comorbidities, n (%)	
Cardiovascular disease	8 (19)

### Summary of Stationary Versus Nonstationary Temperature Asymmetry Signals

**Figure 3.**
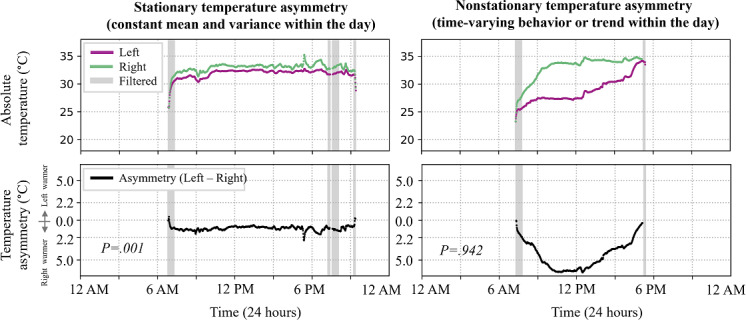
Illustrative stationary (indicating constant mean and variance over time) and nonstationary (indicating time-varying behaviors or trends in the signal) plantar temperature signals. Continuous time series for left (purple) and right (green) plantar temperature measurements (upper row); corresponding left versus right plantar temperature asymmetry (lower row). The gray bars show the masked portions of the time-series signals during the data-cleaning steps. The two illustrative days are from two different participants. *P* values indicate the test result from the augmented Dickey-Fuller test on the temperature asymmetry signal. Signals with a *P* value ≤.05 were considered stationary.

Across the 5400 temperature asymmetry signals analyzed, 82% (n=4428) were nonstationary. Figure 3 shows an illustrative stationary and nonstationary temperature asymmetry signal.

### Stationarity of Temperature Asymmetry Signals in Individual Participants

**Figure 4.**
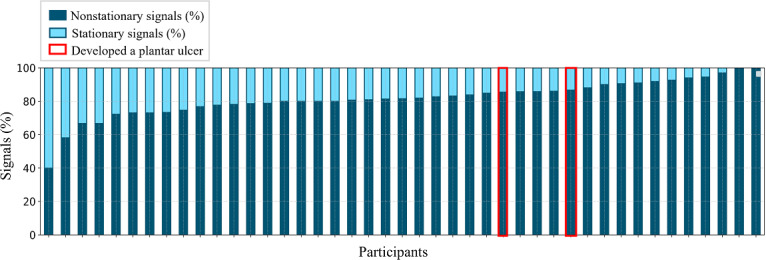
For each participant (n=43), most of the temperature asymmetry signals were nonstationary (indicating time-varying behaviors or trends in the signal). For a given participant, 40% to 100% of their temperature asymmetry signals were nonstationary (dark blue bars) and the remaining signals were stationary (light blue bars). Most of the temperature asymmetry signals were nonstationary for the 2 participants who developed a plantar ulcer (identified with red bars). Participants were visually sorted from lowest to highest percent of nonstationary signals.

For a given participant, 40% to 100% of their temperature asymmetry signals were nonstationary (Figure 4). No participant exhibited entirely stationary data throughout the 90-day study period. For the 2 participants who developed a plantar ulcer in this study, >80% of their temperature asymmetry signals were nonstationary.

### Stationarity of Temperature Asymmetry Signals at Different Plantar Locations

Pooling all participants’ data days, the percentage of nonstationary temperature asymmetry signals was similar at each plantar sensor location: 83% of data days at heel, 82% at Meta 1, 82% at Meta 3, 80% at Meta 5, and 82% at the big toe region.

**Figure 5.**
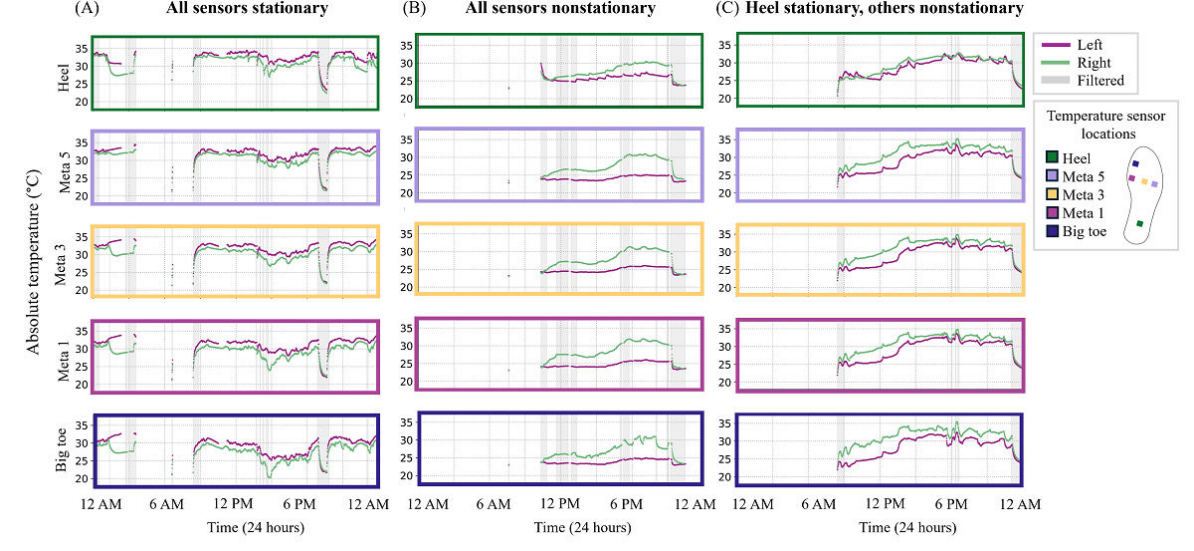
Stationarity of plantar temperature asymmetry signals sometimes varied across sensor locations. Across sensor locations, some data days displayed consistently stationary temperature asymmetry signals (**A**), some displayed consistently nonstationary temperature asymmetry signals (**B**), and others displayed a mix of stationary and nonstationary signals across sensor locations (**C**). The 3 illustrative data days are from 3 different participants.

When analyzing individual participant data, some individuals exhibited a consistent stationarity result across all sensor locations (Figure 5A and B). However, others showed a mix of stationary and nonstationary signals across plantar sensor locations (Figure 5C). For example, in 1 participant, the forefoot sensor (Meta 1, Meta 3, Meta 5, and big toe) signals were nonstationary for >90% of data days, whereas heel temperature asymmetry signals were nonstationary for only 64% of data days. Therefore, this participant often exhibited stationary heel temperature asymmetry while other plantar sensor locations showed nonstationary signals.

### Temperature Trends of Concern in Nonstationary Signals

**Figure 6.**
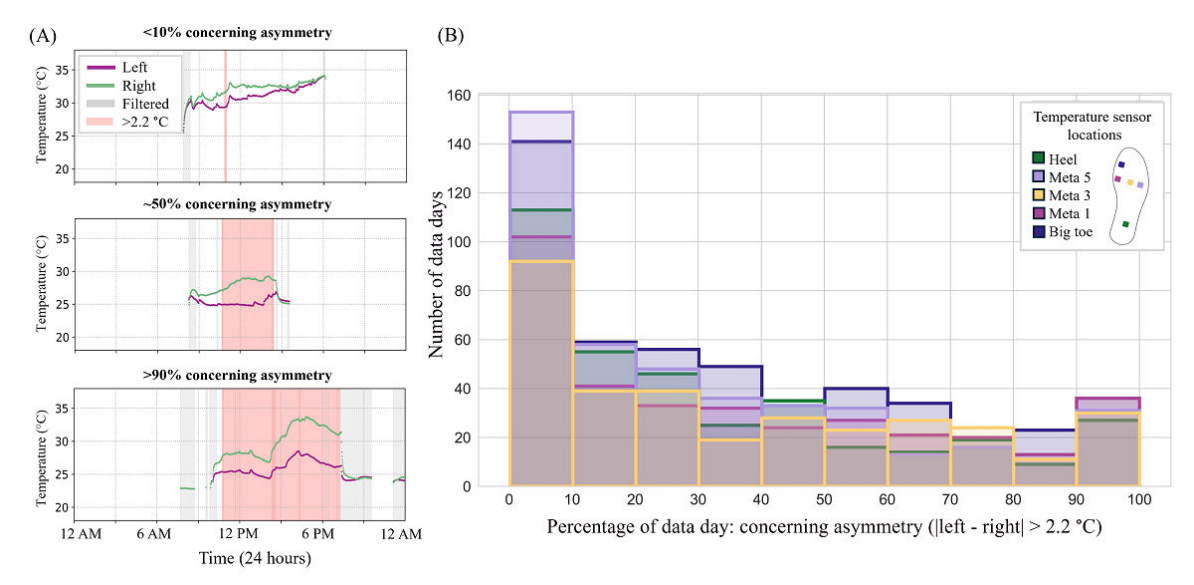
Of the total, 44% of nonstationary signals included one or more concerning temperature asymmetry measurements (>2.2 °C). (**A**) Three illustrative data days showing varying percentage of concerning temperature asymmetry (red shaded region); (**B**) frequency of data days with different percentages of concerning temperature asymmetry measurements at each sensor location.

Of the total, 44% of the nonstationary signals included one or more temperature asymmetry measurements that exceeded a 2.2-°C asymmetry threshold for a data trend of concern. The total number of data days with at least one measurement of concerning asymmetry was 359 for the heel, 431 for Meta 5, 332 for Meta 3, 349 for Meta 1, and 487 for the big toe. The percentage of measurements within a day that exceeded the 2.2-°C threshold varied from very few measurements to nearly all of the daily measurements (Figure 6).

### Temporally Dispersed Concerning Temperature Asymmetry

**Figure 7.**
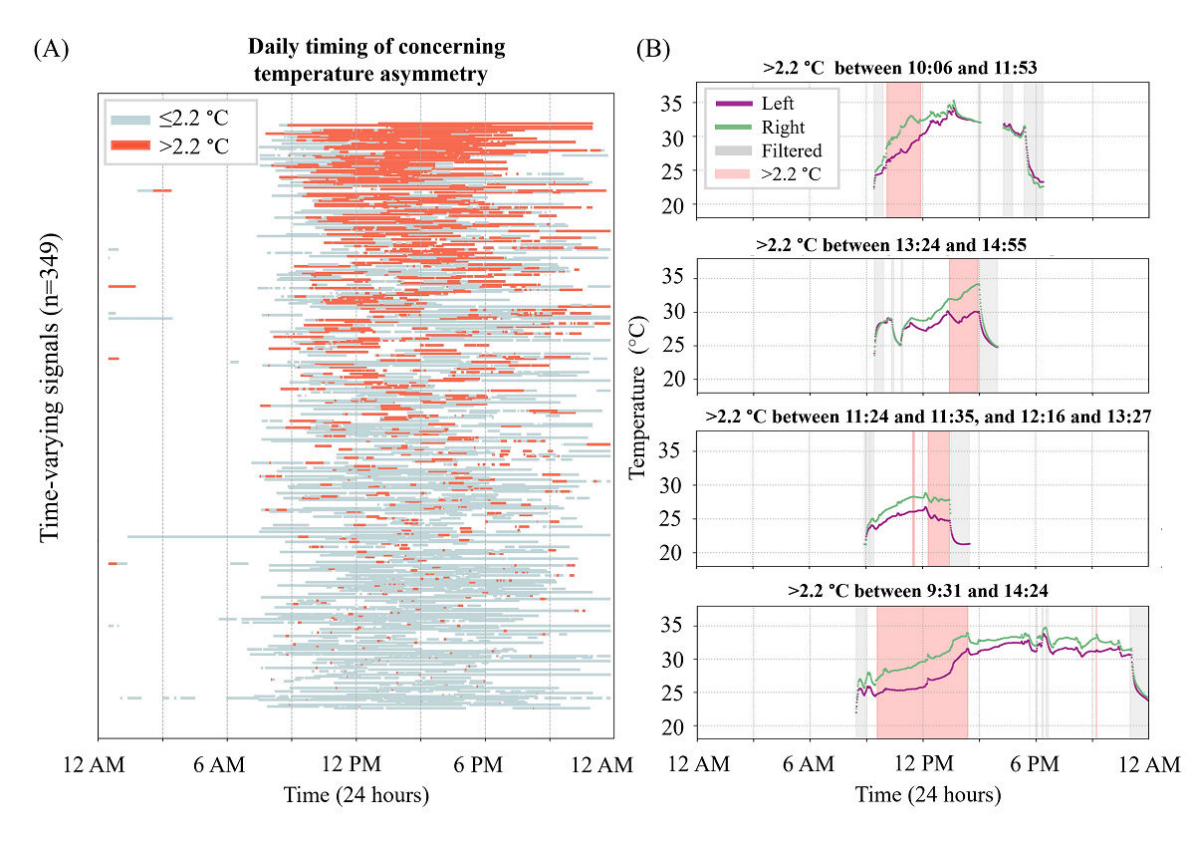
Temporally dispersed concerning temperature asymmetry. (**A**) The distribution of nonconcerning (≤2.2 °C, light blue bands) and concerning (>2.2 °C, red bands) temperature asymmetry measurements across 349 plantar temperature signals that included at least one concerning temperature asymmetry measurement. The results are for one representative sensor location (Meta 1). Each horizontal bar is the usage duration for a data day. The data days were visually ordered based on the duration of the concerning asymmetry. (**B**) Four illustrative data days with at least one concerning temperature asymmetry measurement. Concerning temperature asymmetries occurred at different times of the day.

A visual inspection of nonstationary data days with one or more temperature asymmetry measurements exceeding the 2.2-°C threshold revealed that the periods of concerning temperature asymmetry were temporally dispersed across participants, time windows, and data days (Figure 7A and B).

## Discussion

### Principal Findings

The primary finding of this study was that most (4428/5400, 82%) of the plantar temperature asymmetry signals were nonstationary. Temperature asymmetry fluctuations occurred throughout the day, likely influenced by underlying physiological and environmental factors. About half (1948/4428, 44%) of the nonstationary temperature signals included a mix of concerning (>2.2 °C) and nonconcerning (≤2.2 °C) temperature asymmetry measurements. Single or intermittent measurements of temperature asymmetry are therefore at risk of over- or underestimating plantar hot spots and DFU risk. These results highlight the value of continuous remote temperature monitoring in the management of diabetic foot disease.

### Leveraging Continuous Monitoring to Improve Accuracy

Modest success in DFU risk reduction has been demonstrated with intermittent plantar temperature monitoring [5]. A meta-analysis of 5 randomized controlled trials (n=772) demonstrated that single time-point foot temperature monitoring and reduction of ambulatory activity in response to hot spots (temperature asymmetry >2.2 °C on 2 consecutive days) reduced the risk of developing a DFU by approximately 50% [5]. However, the overall certainty of this evidence was low. Intermittent monitoring protocols may miss transient but clinically relevant changes that occur between measurements.

Of the total, 44% of nonstationary data days included one or more temperature asymmetry measurements that exceeded a 2.2-°C threshold, indicating a data trend of concern. On these days, relying on a single, once-daily snapshot risks both false-negative and false-positive assessments (Figures6  7). Multiple studies have demonstrated the limitations of once-daily temperature monitoring. In a study [7] using a thermometric foot mat to assess once-daily temperature asymmetry (one 20 s measurement), the authors reported a false-positive rate of 57% and false-negative rate of 3% for predicting ulcer development with an average lead time of 37 days. Another study [25] found that in the 2 months preceding ulceration, only 8 (28%) participants had a true hot spot, while 7 (24%) had false hot spots and 14 (48%) had no detectable hot spot, challenging the assumption that once-daily measurements consistently identify impending skin breakdown. Similarly, a post hoc analysis [26] of foot thermograms from 15 individuals with diabetes identified hot spots (asymmetry >2.2 °C) in 20% of regions of interest, yet none of the participants developed ulcers. While several factors likely contribute to the high rate of false positives and false negatives reported, one explanation may be that single snapshot measurements fail to capture the inherent variability of intraday plantar temperature asymmetries.

Several repeated measurements throughout the day, rather than a once-daily measurement, increase confidence with regard to the accuracy of observed plantar physiology trends. The recommended frequency of daily plantar temperature measurements may need to be increased to provide a more holistic view of a patient’s foot health.

### Uncovering New Diagnostic Opportunities

Across participants, 40% to 100% of temperature asymmetry signals were nonstationary (Figure 4), and individual participants displayed different distributions of stationary versus nonstationary signals at each plantar location (Figure 5). Numerous factors such as activity, foot care practices, vascular disease status, history of ulceration, foot posture and gait, and foot deformities may contribute to the observed temperature signal patterns [19,27]. While current remote temperature monitoring protocols emphasize the *magnitude* of temperature asymmetry, it is possible the *pattern* of temperature asymmetry also offers diagnostic value. As a secondary, exploratory analysis, we examined 3 illustrative participants to explore potential associations between participant-level factors and patterns of temperature asymmetry.

**Figure 8.**
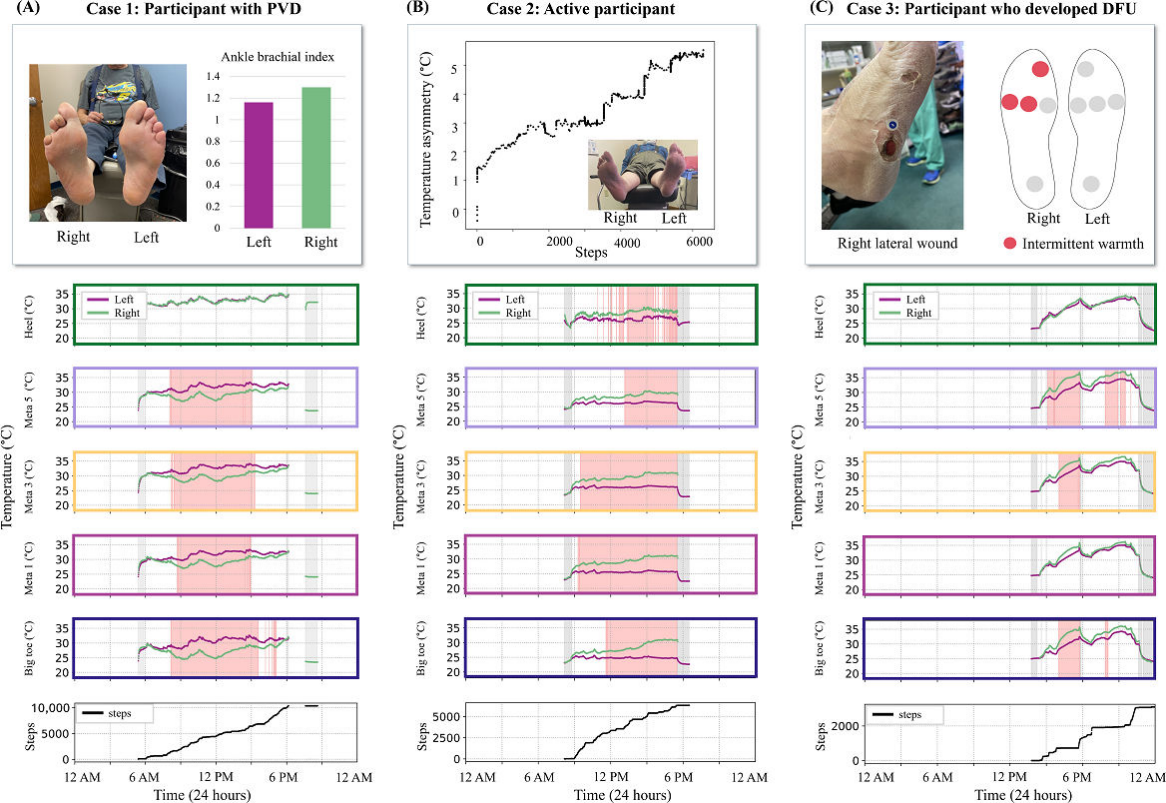
Three illustrative participants to demonstrate potential associations between participant-level factors and patterns of temperature asymmetry. DFU: diabetic foot ulcer; PVD: peripheral vascular disease.

Case 1 is a participant with comorbid peripheral vascular disease. Continuous plantar temperature monitoring may be particularly valuable for individuals with peripheral vascular disease, a common comorbidity of diabetes, as limb temperature can also serve as a surrogate measure of limb perfusion [28]. The most recent vascular assessment for this participant (approximately 4 mo before enrollment in the study) showed a right ABI of 1.3, suggesting arterial calcification and noncompressibility, and a left ABI measurement of 1.16, within the normal range (Figure 8A). Interestingly, the sensory insole typically measured lower temperatures in forefoot regions on the right foot, aligning with the presumed calcification in that limb. Temperature asymmetry was nonstationary at the forefoot sensor sites (Meta 1, Meta 3, Meta 5, and big toe) but not at the heel. This pattern may reflect the impact of greater vessel length supplying the forefoot compared to the heel, which is served by separate, more proximal arterial branches. It should be noted that this participant was excluded from the primary study analysis due to a right fourth toe amputation (Figure 2), but it is reported here as an exploratory example to illustrate the potential relationship between vascular disease status and temperature asymmetry profile.

Case 2 is a participant with bilateral hammertoes and high daily step counts. The participant exhibited nonstationary temperature asymmetry patterns at all foot regions (5 sensor locations), with the right limb typically presenting as warmer. Interestingly, the magnitude of temperature asymmetry increased progressively with time and cumulative steps throughout the day (Figure 8B). We suspect that the correlation between temperature asymmetry and activity may reflect asymmetrical loading or altered biomechanics from the underlying foot deformities, potentially leading to increased tissue stress and localized inflammation on the right side. This participant was not reported to have underlying vascular disease.

Case 3 is a participant who developed an ulcer at the lateral aspect of the right foot during the study. Notably, the participant exhibited nonstationary temperature asymmetry patterns with episodes of temperature asymmetry exceeding the concerning threshold of 2.2 °C intermittently throughout the day (Figure 8C). The location of intermittent warmth (Meta 3, Meta 5, and big toe) did not exactly correspond to the reported ulcer location (ie, the lateral aspect of the foot). However, these transient temperature elevations, with the right forefoot being relatively warmer, would have been at risk of being missed by once-daily temperature assessments, highlighting the advantage of continuous intraday temperature monitoring. No meaningful data trends were observed for the other participant who developed an ulcer during the study, likely due to poor adherence to the sensory insoles, which were worn for only 3 days.

Exploratory observations from these representative cases highlight the potential for continuous temperature monitoring to offer insights that go far beyond those provided by once-daily measurements. We hypothesize that the ongoing collection of multimodal, continuous plantar data will uncover meaningful associations and previously unrecognized patterns that link temperature asymmetry with vascular status, activity, wound risk, or other factors. Continuous data may enable more personalized and timely interventions based on when and where risk emerges.

### A Step Toward Personalized Monitoring

Once-daily foot skin temperature monitoring is a clear, concise, and achievable guideline [7]. However, such one-size-fits-all guidelines may not be appropriate for all patients. In this study, a review of all data days that included one or more measurements exceeding the threshold of concern suggested there was not a consistent time of day when concerning temperature asymmetries typically occurred (Figure 7). A fixed, single time point for monitoring temperature asymmetry risks under- or overestimating risk.

Greater visibility into the day-by-day temperature asymmetry fluctuations may be meaningful to providers and patients, similar to how continuous glucose monitoring information and a core set of glucose level statistics overcame the limited information provided by isolated glucose readings [29]. Such continuous monitoring may better support health care providers to personalize care for the individual. We anticipate that continuous temperature asymmetry signals will be used increasingly in clinical trials for managing diabetic foot health.

### Limitations and Future Opportunities

Many unknowns remain regarding the underlying physiological and environmental factors that contribute to intraday fluctuations in plantar temperature asymmetry. Additional clinical trials are necessary to determine the degree to which continuous temperature monitoring improves DFU detection compared to once-daily temperature measurements. While ongoing research is necessary to better understand these mechanisms, we remain confident that continuous, repeated measurements throughout the day build more confidence for clinical decision-making.

Further research is needed to determine if and how plantar temperature warning thresholds should be adapted for continuous monitoring. Current guidelines, based on clinical trials using once- or twice-daily measurements, recommend a hot spot indicator threshold of 2.2 °C for 2 or more consecutive days. However, with the recent availability of continuous plantar temperature monitoring, it may be necessary to reconsider this fixed threshold. A variable or individualized threshold, accounting for factors such as activity levels, comorbid peripheral vascular disease, previous ulcer locations, and environmental influences, may be more appropriate.

Moreover, continuous temperature monitoring provides an opportunity to move beyond the static threshold-based criteria to other statistical strategies, such as time in range versus time above threshold, intraday trend and variability metrics, or interday temperature profile comparisons. This evolution parallels the development of extended analytics during the transition from discrete self-monitoring of blood glucose to continuous glucose monitoring systems [16,17]. Developing updated reporting standards will require further research and multidisciplinary collaboration to achieve consensus. Ultimately, we envision a future in which continuous temperature monitoring and its associated analytics become as ubiquitous and clinically valuable as continuous glucose monitoring and other continuous physiologic monitoring tools.

### Conclusions

This study demonstrates the high prevalence (82% of data days) of nonstationary temperature asymmetry signals in individuals with diabetes and history of ulceration. Our results highlight the value of continuous plantar temperature monitoring for diabetic foot health management, relative to once-daily measurements. Continuous monitoring may improve the accuracy of plantar temperature measurement, unlock new diagnostic capabilities, and support personalized monitoring. Future research is required to better understand the underlying mechanisms of plantar temperature asymmetry fluctuations and to prove the clinical value of continuous monitoring for reducing the risk of DFU formation. Continuous monitoring is an exciting advancement in remote temperature monitoring for diabetes health span extension.

## Supplementary material

10.2196/73187 Checklist 1 STROBE reporting checklist.

## Abbreviations:

ABI: ankle-brachial index
ADF: augmented Dickey-Fuller
AIC: Akaike information criterion
CAD/CAM: computer-aided design/computer-aided manufacturing
DFU: diabetic foot ulcer
IMU: inertial measurement unit
STROBE: Strengthening the Reporting of Observational Studies in Epidemiology
