# Supplementary material for: Exploring the Value of Continuous Plantar Temperature Monitoring for Diabetic Foot Health Management: Observational, Prospective Cohort Study
Source: JMIR Mhealth Uhealth. 2025 Sep 12;13:e73187. doi: 10.2196/73187 (PMC12431787; doi:10.2196/73187)
Supplement: Checklist 1 [file mhealth-v13-e73187-s001.docx]

STROBE (Strengthening the Reporting of Observational Studies in Epidemiology) reporting checklist.

|  | Item No. | Recommendation | Page  No. | Author notes |
| --- | --- | --- | --- | --- |
| **Title and abstract** | 1 | (*a*) Indicate the study’s design with a commonly used term in the title or the abstract  (*b*) Provide in the abstract an informative and balanced summary of what was done and what was found | 1 | Exploring the Value of Continuous Plantar Temperature  Monitoring for Diabetic Foot Health Management:  Observational, Prospective Cohort Study |
| Introduction | | | |  |
| Background/rationale | 2 | Explain the scientific background and rationale for the investigation being reported | 2 |  |
| Objectives | 3 | State specific objectives, including any prespecified hypotheses | 2 | Given the novel nature of this analysis, no prespecified hypotheses were set. Rather, the study aimed to characterize signal patterns and assess their stationarity using statistical methods. |
| Methods | | | |  |
| Study design | 4 | Present key elements of study design early in the paper | 2-3 |  |
| Setting | 5 | Describe the setting, locations, and relevant dates, including periods of recruitment, exposure, follow-up, and data collection | 2-3 |  |
| Continued on next page |  |  |  |  |
| Participants | 6 | (*a*) *Cohort study*—Give the eligibility criteria, and the sources and methods of selection of participants. Describe methods of follow-up  (*b*) *Cohort study*—For matched studies, give matching criteria and number of exposed and unexposed | 2-3 | This was an unmatched study; no matching criteria were applied. |
| Variables | 7 | Clearly define all outcomes, exposures, predictors, potential confounders, and effect modifiers. Give diagnostic criteria, if applicable | 3-4 |  |
| Data sources/ measurement | 8* | For each variable of interest, give sources of data and details of methods of assessment (measurement). Describe comparability of assessment methods if there is more than one group | 3-4 |  |
| Bias | 9 | Describe any efforts to address potential sources of bias | 3 | Several participant and signal exclusion criteria specific to this analysis are described that aim to minimize potential sources of bias |
| Study size | 10 | Explain how the study size was arrived at | N/A | No formal power analysis was conducted to determine the sample size. As a prospective observational study, our aim was to enrol all eligible participants during the defined recruitment period based on resource availability. The sample size was therefore determined pragmatically rather than by a prior statistical analysis. |
| Quantitative variables | 11 | Explain how quantitative variables were handled in the analyses. If applicable, describe which groupings were chosen and why | 3-4 |  |
| Continued on next page |  |  |  |  |
| Statistical methods | 12 | (*a*) Describe all statistical methods, including those used to control for confounding  (*b*) Describe any methods used to examine subgroups and interactions  (*c*) Explain how missing data were addressed  (*d*) *Cohort study*—If applicable, explain how loss to follow-up was addressed  (*e*) Describe any sensitivity analyses | 3-4 |  |
| Results |  |  |  |  |
| Participants | 13* | (a) Report numbers of individuals at each stage of study—eg numbers potentially eligible, examined for eligibility, confirmed eligible, included in the study, completing follow-up, and analysed  (b) Give reasons for non-participation at each stage  (c) Consider use of a flow diagram | 4, Figure 2 |  |
| Descriptive data | 14* | (a) Give characteristics of study participants (eg demographic, clinical, social) and information on exposures and potential confounders  (b) Indicate number of participants with missing data for each variable of interest  (c) *Cohort study*—Summarise follow-up time (eg, average and total amount) | 4, Table 1 |  |
| Outcome data | 15* | *Cohort study*—Report numbers of outcome events or summary measures over time | 5-8 |  |
| Main results | 16 | (*a*) Give unadjusted estimates and, if applicable, confounder-adjusted estimates and their precision (eg, 95% confidence interval). Make clear which confounders were adjusted for and why they were included  (*b*) Report category boundaries when continuous variables were categorized  (*c*) If relevant, consider translating estimates of relative risk into absolute risk for a meaningful time period | 5-8, Figure 3-7 | Statistical means were not reported, therefore confidence intervals were not included. |
| Other analyses | 17 | Report other analyses done—eg analyses of subgroups and interactions, and sensitivity analyses | N/A | No sub-group analyses |
| Discussion |  |  |  |  |
| Key results | 18 | Summarise key results with reference to study objectives | 8-10, Figure 8 |  |
| Limitations | 19 | Discuss limitations of the study, taking into account sources of potential bias or imprecision. Discuss both direction and magnitude of any potential bias | 10-11 |  |
| Continued on next page |  |  |  |  |
| Interpretation | 20 | Give a cautious overall interpretation of results considering objectives, limitations, multiplicity of analyses, results from similar studies, and other relevant evidence | 8-11 |  |
| Generalisability | 21 | Discuss the generalisability (external validity) of the study results | 8-11 |  |
| Other information |  |  |  |  |
| Funding | 22 | Give the source of funding and the role of the funders for the present study and, if applicable, for the original study on which the present article is based | 11 |  |
